# The genetic relationship between educational attainment and cognitive performance in major psychiatric disorders

**DOI:** 10.1038/s41398-019-0547-x

**Published:** 2019-08-28

**Authors:** Ashley L. Comes, Fanny Senner, Monika Budde, Kristina Adorjan, Heike Anderson-Schmidt, Till F. M. Andlauer, Katrin Gade, Maria Hake, Urs Heilbronner, Janos L. Kalman, Daniela Reich-Erkelenz, Farah Klöhn-Saghatolislam, Sabrina K. Schaupp, Eva C. Schulte, Georg Juckel, Udo Dannlowski, Max Schmauß, Jörg Zimmermann, Jens Reimer, Eva Reininghaus, Ion‐George Anghelescu, Volker Arolt, Bernhard T. Baune, Carsten Konrad, Andreas Thiel, Andreas J. Fallgatter, Vanessa Nieratschker, Christian Figge, Martin von Hagen, Manfred Koller, Thomas Becker, Moritz E. Wigand, Markus Jäger, Detlef E. Dietrich, Sebastian Stierl, Harald Scherk, Carsten Spitzer, Here Folkerts, Stephanie H. Witt, Franziska Degenhardt, Andreas J. Forstner, Marcella Rietschel, Markus M. Nöthen, Jens Wiltfang, Peter Falkai, Thomas G. Schulze, Sergi Papiol

**Affiliations:** 1 Institute of Psychiatric Phenomics and Genomics, University Hospital, LMU Munich, Munich, 80336 Germany; 2 International Max Planck Research School for Translational Psychiatry (IMPRS-TP), Munich, 80804 Germany; 3 Department of Psychiatry and Psychotherapy, University Hospital, LMU Munich, Munich, 80336 Germany; 4 Department of Psychiatry and Psychotherapy, University Medical Center Göttingen, Göttingen, 37075 Germany; 5 Department of Neurology, Klinikum rechts der Isar, School of Medicine, Technical University of Munich, Munich, 81675 Germany; 6 Department of Psychiatry, Ruhr University Bochum, LWL University Hospital, Bochum, 44791 Germany; 7 Department of Psychiatry, University of Münster, Münster, 48149 Germany; 8 Department of Psychiatry and Psychotherapy, Bezirkskrankenhaus Augsburg, Augsburg, 86156 Germany; 9 Psychiatrieverbund Oldenburger Land gGmbH, Karl-Jaspers-Klinik, Bad Zwischenahn, 26160 Germany; 10 Department of Psychiatry and Psychotherapy, University Medical Centre Hamburg-Eppendorf, Martinistr. 52, Hamburg, 20246 Germany; 11 Department of Psychiatry and Psychotherapeutic Medicine, Research Unit for Bipolar Affective Disorder, Medical University of Graz, Graz, 8036 Austria; 12 Department of Psychiatry, Dr. Frontheim-Mental Health, Liebenburg, 38704 Germany; 13 Department of Psychiatry, Melbourne Medical School, The University of Melbourne, Melbourne, VIC, 3010 Australia; 14 Department of Psychiatry and Psychotherapy, Agaplesion Diakonieklinikum, Rotenburg, 27356 Germany; 15 Department of Psychiatry and Psychotherapy, University of Tübingen, Tübingen, 72076 Germany; 16 Karl-Jaspers Clinic, European Medical School Oldenburg-Groningen, Oldenburg, 26160 Germany; 17 Clinic for Psychiatry and Psychotherapy, Clinical Center Werra-Meißner, Eschwege, 37269 Germany; 18 Asklepios Specialized Hospital, Göttingen, 37081 Germany; 19 Department of Psychiatry II, Ulm University, Bezirkskrankenhaus Günzburg, Günzburg, 89312 Germany; 20 AMEOS Clinical Center Hildesheim, Hildesheim, 31135 Germany; 21 Center für Systems Neuroscience (ZSN) Hannover, Hannover, 30559 Germany; 22 Dept. of Psychiatry, Medical School of Hannover, Hannover, 30625 Germany; 23 Psychiatric Hospital Lüneburg, Lüneburg, 21339 Germany; 24 AMEOS Clinical Center Osnabrück, Osnabrück, 49088 Germany; 25 ASKLEPIOS Specialized Hospital Tiefenbrunn, Rosdorf, 37124 Germany; 26 Department of Psychosomatic Medicine, University Medicine Rostock, Rostock, 18051 Germany; 27 Department of Psychiatry, Psychotherapy and Psychosomatics, Clinical Center Wilhelmshaven, Wilhelmshaven, 26389 Germany; 28 Department of Genetic Epidemiology in Psychiatry, Central Institute of Mental Health, Medical Faculty Mannheim, University of Heidelberg, Mannheim, 68159 Germany; 29 Institute of Human Genetics, University of Bonn School of Medicine & University Hospital Bonn, Bonn, 53127 Germany; 30 Center for Human Genetics, University of Marburg, Marburg, 35033 Germany; 31 Department of Biomedicine, University of Basel, Basel, 4031 Switzerland; 32 Department of Psychiatry (UPK), University of Basel, Basel, 4002 Switzerland; 33 German Center for Neurodegenerative Diseases (DZNE), Göttingen, 37075 Germany; 34 iBiMED, Medical Sciences Department, University of Aveiro, Aveiro, 3810-193 Portugal

**Keywords:** Genomics, Bipolar disorder, Schizophrenia, Clinical genetics, Learning and memory

## Abstract

Cognitive deficits are a core feature of psychiatric disorders like schizophrenia and bipolar disorder. Evidence supports a genome-wide polygenic score (GPS) for educational attainment (GPS_EDU_) can be used to explain variability in cognitive performance. We aimed to identify different cognitive domains associated with GPS_EDU_ in a transdiagnostic clinical cohort of chronic psychiatric patients with known cognitive deficits. Bipolar and schizophrenia patients from the PsyCourse cohort (*N* = 730; 43% female) were used. Likewise, we tested whether GPSs for schizophrenia (GPS_SZ_) and bipolar disorder (GPS_BD_) were associated with cognitive outcomes. GPS_EDU_ explained 1.5% of variance in the backward verbal digit span, 1.9% in the number of correctly recalled words of the Verbal Learning and Memory Test, and 1.1% in crystallized intelligence. These effects were robust to the influences of treatment and diagnosis. No significant associations between GPS_SZ_ or GPS_BD_ with cognitive outcomes were found. Furthermore, these risk scores did not confound the effect of GPS_EDU_ on cognitive outcomes. GPS_EDU_ explains a small fraction of cognitive performance in adults with psychiatric disorders, specifically for domains related to linguistic learning and working memory. Investigating such a proxy-phenotype longitudinally, could give intriguing insight into the disease course, highlighting at what time genes play a more influential role on cognitive performance. Better understanding the origin of these deficits might help identify those patients at risk for lower levels of functioning and poor social outcomes. Polygenic estimates may in the future be part of predictive models for more personalized interventions.

## Introduction

Cognitive deficits are a core and robust feature of psychiatric disorders like bipolar disorder and schizophrenia, present even during periods of remission^1–3^. These deficits are key predictors of long-term functional and social outcomes and are difficult to treat with current pharmaceutical options or behavioral interventions^4–6^. Considering the associated psychosocial burden and high prevalence of these deficits among patients, psychiatric researchers have put considerable effort towards understanding their underlying mechanisms. Thus far, genome-wide association studies (GWAS) have provided evidence supporting the polygenic architecture and remarkable heritability of cognitive performance in population-based cohorts^7–10^. Furthermore, evidence supports the phenotypic and genetic stability of individual cognitive differences across the lifetime in domains including executive functioning, attention, and verbal memory^11–14^. As studies have shown evidence of impairments even in unaffected first-degree relatives of individuals with psychiatric disorders, cognitive deficits have been hypothesized as a valuable endophenotype of interest for better understanding the genetic risk factors of psychiatric disorders^15–17^.

Intelligence, encompassing cognition, is highly heritable and an imperative predictor of occupational and health outcomes^18^. Despite high heritability estimates of intelligence, indicated to be up to 80% in adulthood^19^, unraveling the underlying genetic contribution of intelligence differences using GWAS has been challenging and thus far little of the observed heritability has been explained^11,20^. To date, studies on intelligence have been limited by insufficient sample sizes and further complicated by the challenge of precise and reliable measurements for this complex phenotype^7–10,20^. The latest GWAS on intelligence identified 205 genomic loci implicating up to 1016 genes, which explained approximately five percent of the variance in intelligence^21^. Another large study reported a genome-wide polygenic score (GPS) that could explain 4.3% of variance in general cognitive function^11^.

Educational attainment is moderately heritable and has been obtained as a demographic item in countless medical datasets and for cohorts of which genetic data is available^22^. In the last decade, educational attainment has been proposed as a proxy-phenotype for cognition, as it is highly associated with intelligence both on a phenotypic (0.50) and genetic level (0.65)^18,20,23–26^. Notably, GPS based on GWAS summary statistics for years of education predict more variance in intelligence than the phenotype years of education per se^18,25^, reflecting the substantial genetic correlation between both phenotypes. The largest GWAS of educational attainment published to date, based on 1.1 million individuals, identified 1271 lead single nucleotide polymorphisms (SNPs)^22^. Through a multi-phenotype analysis of educational attainment and three cognitive phenotypes, the authors were able to generate a GPS which explained 7–10% of variance in cognitive performance in the general population. The SNPs identified implicated genes involved in neurodevelopmental processes and neuron-to-neuron communication^22^. The authors showed that the use of educational attainment as a proxy-phenotype could uncover genetic variants to be used as a set of “empirically-based candidate genes” for future studies, for example testing associations with important endophenotypes like cognition^27^.

Studies have already demonstrated an important association of educational attainment GPS (GPS_EDU_) with cognitive performance, showing that, in a general population, a higher GPS is associated with higher performance on neurocognitive tests^28^. However, limited evidence exists supporting this association in patients with known cognitive deficits^29,30^, and there remains a need to investigate this association across different cognitive domains. Here we analyzed whether GPS_EDU_ could be used to explain variability in different cognitive domains in chronic patients with schizophrenia and bipolar disorder from the PsyCourse cohort^31^. This transdiagnostic approach aligns with the growing evidence for dimensional models that cut across diagnostic categories in psychiatry and is supported by the large cognitive, clinical and genetic overlaps between both disorders^32,33^. Particularly, the genetic overlap between both disorders has been firmly established by heritability estimates derived from population-based multi-generation registers^34^ and by recent molecular studies that have reported an outstanding genetic correlation (*r*_*g*_ = 0.70 ± 0.02)^35^.

Considering the positive genetic correlations reported between education and both schizophrenia (*r*_*g*_ = 0.10) and bipolar risk (*r*_*g*_ = 0.28)^36^, we further assessed how GPSs for both schizophrenia (GPS_SZ_) and bipolar disorder (GPS_BD_) were associated with cognitive performance in our sample.

## Materials & methods

### Participants

Data were used from the multicenter, PsyCourse study in Germany and Austria, consisting of participants of European ancestry (www.PsyCourse.de)^31^. Participants were phenotyped using a comprehensive battery including data on socio-demographics, psychopathology, cognition, and functioning assessed at each of four visits (6-month intervals). Recruitment strategies and characterization of all participants has been previously described in detail^31^. The sample selected for this project comprised a total of 730 participants with a DSM-IV^37^ diagnosis of schizophrenia, schizoaffective disorder, or bipolar disorder (type I or II). Additionally, cognitive data available from 320 nonclinical (control) participants was used to give an orientation to the range of phenotypic data available in the PsyCourse cohort and to confirm general, well-replicated findings of lower cognitive performance in patients with psychiatric disorders compared to healthy controls. The study was approved by the local ethics committee for each study center and was carried out following the rules of the Declaration of Helsinki. All individuals provided written informed consent as previously described^31^.

### Psychopathology psychometric instrument

The Positive and Negative Syndrome Scale (PANSS) is a clinical instrument used to measure symptom severity in schizophrenia and routinely used to assess a variety of disorders including bipolar disorder^38^. A continuous, total score of the three subscales, i.e., positive (e.g., hallucinations and delusions), negative (e.g., emotional withdrawal and blunted affect), and general symptoms (e.g., somatic concern and poor attention) was used as an indication of disease severity at the time of testing.

### Cognitive performance psychometric instruments

Cognitive tests were administered at each study visit. The Verbal Learning and Memory Test (VLMT) was introduced at visit 2. For all other cognitive measures, scores from visit 1 were used for analyses.

#### Crystallized intelligence

The MWT–B (Mehrfachwahl–Wortschatz–Intelligenz test) was used to measure crystallized intelligence^39,40^. In this test, subjects were presented with 37 sets of five words arranged according to the level of difficulty. Four words of each set were fictitious constructions of known vernacular (i.e., they do not exist in the German language), while one word really exists. Subjects were asked to cross out the word they know to exist. The total number of correctly marked lines was used as a score^40^.

#### Trail-Making-Test (TMT)

The TMT is a measure of visual attention and task switching and is one of several executive functioning measures. The test consists of two parts, part A assesses psychomotor speed of the participant, and part B assesses switching between two automated tasks (counting and reciting the alphabet). The time taken to complete each part of the test was measured and the difference in time needed (part B-part A) was used, as it is considered a more accurate measure of the divided attention and alternating sequencing tasks tested in part B^41–43^. In this case, a higher score meant worse cognitive performance.

#### Verbal digit span

The verbal digit span, from the Wechsler Adult Intelligence Scale, assesses short-term (forward digit-span) and working memory capacity (backward digit-span). Briefly, participants were asked to recall verbally a sequence of digits, with increasingly longer sequences in each trial. For each correctly recalled string of digits, one point was given. The test was ended when the participant was unable to correctly repeat two presented strings of the same length. The difference between the forward and backward task is that the latter involves mental manipulation as the participant is required to repeat the digits in backward order^44^. A score for each task was considered.

#### Digit-Symbol-Test (DST)

The DST is a subset of the Wechsler Adult Intelligence Scale^45^ and measures processing speed, working memory, visuospatial processing and attention. In this test, the participant was asked to use a key of numbers 1–9 with coinciding symbols to draw the appropriate symbol that matched the number given. The participant was given 120 s to fill in as many corresponding symbols as possible. In the end, the correct number of symbols drawn was totaled to get an overall score.

#### Verbal Learning and Memory Test (VLMT)

The VLMT is the German version of the Auditory Verbal Learning Test^46^. This word-list learning paradigm assesses several memory parameters through serial list learning with subsequent distraction, retrieval after distraction and half-hour time delay, and through a recognition task. The test consists of two different word lists which are each 15 independent words and a recognition list which includes 30 words from the two lists and 20 similar distractor words. Four VLMT scores were rated, the first for the number of correctly recalled words from the first list, a second score for the number of words lost after distraction, a third score of words lost after a time interval, and a fourth score of correctly recalled words from the recognition list^47^.

### Biological samples

Peripheral blood samples were used for DNA extraction using standard techniques. DNA samples were then used to genotype patients for calculation of GPSs. Genotype data for controls was not available at the time of this investigation and they have not been used for GPS analyses.

### GPS estimation

DNA samples were genotyped using the Infinium PsychArray Beadchip (Psychip, Illumina, San Diego, CA, USA). Following standard quality control procedures, imputation was performed using the 1000 Genomes Phase 3 reference panel as previously described in detail^31,48^. GPSs were calculated for all individuals using PLINK 1.90b5.3^49^. Summary statistics for educational attainment were obtained from the Social Science Genetic Association Consortium (https://www.thessgac.org/data)^22^. These summary stats are derived from analyses excluding 23andMe samples. Summary statistics from the most recent Psychiatric Genomics Consortium GWASs for schizophrenia^50^ and bipolar disorder^51^ were used. All GPSs were calculated based on summary statistics from the discovery datasets, excluding low quality imputed variants (info score < 80%) in the test dataset, rare SNPs (minor allele frequency < 0.05), and ambiguous markers (A/T and C/G). Following the methodology of previous studies^52^, SNPs in the extended major histocompatibility complex region (chromosome 6: 25–34 Mbp) were completely excluded for the calculation of GPS_EDU_ while only the top-associated SNP in this region was included for the calculations of GPS_SZ_ and GPS_BD_. Data was clumped in windows of 500 kbp, discarding variants in LD (*R*^*2*^ > .1) with another more significantly associated marker.

GPSs were then calculated by multiplying the imputation dosage for each risk allele by the log(OR) of each genetic variant. The resulting values were summed to obtain an individual estimate of the genetic burden in each individual across different SNP *p*-value thresholds (*p*_T_). Scores for GPS_SZ_ and GPS_BD_ were calculated based on best discrimination thresholds according to previous findings, i.e., *p*_T_ < 0.05^50^ and *p*_T_ < 0.01^51^, respectively. GPS_EDU_ was calculated at four different *p*-value thresholds, from including only genome-wide significant SNPs to inclusion of all SNPs: *p*_T_ < 5 × 10^−8^, 0.05, 0.1, and 1. All GPSs were approximately normally distributed and standardized via z-score transformation.

### Statistical analyses

#### Sample characteristics

As proof of concept, the effect of case status on cognitive performance was investigated using participants from the PsyCourse cohort. Visual inspection of boxplots comparing case versus control scores was performed and the effect of case status on cognitive domains was further determined through linear regression models, adjusting for age and sex. Socio-demographic and clinical characteristics were tested for between-group differences using the independent sample t-test for continuous data and Pearson’s chi-squared tests for categorical variables. As an additional validation analysis, we investigated the relationship between GPS_EDU_ and educational attainment in our sample using ordinal logistic regression, adjusting for age, sex, the interaction between age and sex, and the first 10 PCs, according to previous work^22^. All analyses were performed using R statistical software version 3.4.0^53^. An initial examination of the distributions of raw cognitive scores was performed to identify and exclude outliers based on Tukey’s definition (removal of values beyond 3× the interquartile range)^54^.

#### GPS analyses of cognitive performance

The effect of GPS_EDU_ on cognitive performance of cases was explored. Blockwise linear regression models were used to estimate the amount of variation in cognitive performance explained by the z-standardized GPS_EDU_ at the four thresholds previously described. For each cognitive outcome, all base models were adjusted for confounding variables measured at the time of testing, i.e., age, age^2^, sex, in/outpatient status, study center, and PANSS sum scores. Although our participants are chronic patients, duration of illness was considered an important covariate which could confound our results. However, as duration of illness proved to be well correlated with age (*r* = 0.53), ultimately only age was kept in the models. To guard against population stratification, the first 20 ancestry principal components (PCs) were included in our models and selected for each cognitive outcome tested using backward model selection (*p* < 0.05)^55^. The significant PCs were as follows: PCs 12 and 17 for the Verbal digit span (forward task); PC 7 for the DST; PCs 1 and 18 for the MWT-B; PCs 1 and 5 for the VLMT- loss of words after time; and PC 16 for the VLMT- correctly recognized words. No significant PCs were found for the other cognitive outcomes. For each cognitive outcome of interest, we measured the incremental adjusted-*R*^*2*^, that is the gain in the coefficient of determination when the GPS_EDU_ was added as covariate to the regression model for each phenotype (cognition score) on a set of baseline covariates. Multiple testing was corrected for using the False Discovery Rate (FDR) method correcting for the polygenic profiles at all four thresholds and for all phenotypes investigated. Visual inspection of the residuals for each model was performed to be sure the requirement of normally distributed model residuals had been fulfilled.

#### GPS analyses of schizophrenia and bipolar disorder

Using blockwise linear regression models as described above, we tested whether polygenic scores for schizophrenia and bipolar disorder influenced cognitive outcomes. This was tested for both the GPS_SZ_ and GPS_BD_ separately. We then determined how the genetic risk for schizophrenia and bipolar disorder influenced the effect of GPS_EDU_ on cognitive outcomes. Both scores were included (separately) in those models in which GPS_EDU_ was significantly associated with the cognitive outcome tested.

#### Additional analyses

Post hoc analyses were performed to determine the robustness of our findings when correcting for diagnosis (bipolar-I disorder, bipolar-II disorder, schizophrenia, or schizoaffective disorder) and medication (number of antipsychotics, antidepressants, mood stabilizers, and tranquilizers at time of assessment). Furthermore, taking into consideration the significant correlation between memory and crystallized intelligence^56^, we performed a mediation analysis introducing the DST and VLMT (number of correctly recalled words) as covariates in our model testing the association between GPS_EDU_ (*p*_T_ < 1) and crystallized intelligence. Multicollinearity diagnostics were performed.

## Results

A description of socio-demographic variables for participants is presented in Table 1. Seven-hundred and thirty patients with schizophrenia and bipolar disorder were used for analyses. The mean age of these participants was 43.19 years, the majority of which were male. The majority of cases (46.2%) were diagnosed with schizophrenia, 10.0% were schizoaffective, 35.1% were bipolar-I patients and 8.7% were bipolar-II patients. During baseline visits, 47.7% of patients were being treated as day/inpatients.

**Table 1:** Sample characteristics

	Cases (*n* = 730)^b^	Controls (*n* = 320)^b^	Test statistic	Degrees of freedom (df)	*p*-value
**Age at baseline**	43.19 (13.01)	37.53 (15.83)	5.62	516.11	<0.001
**Sex**			24.22	1	<0.001
Male	414 (56.7)	128 (40.0)			
**Diagnosis**		N/A	N/A	N/A	N/A
Schizophrenia	337 (46.2)				
Schizoaffective	73 (10.0)				
Bipolar-I disorder	256 (35.1)				
Bipolar-II disorder	64 (8.7)				
**Education** ^a^			154.11	6	<0.001
0	10 (1.4)	0 (0.0)			
1	46 (6.3)	2 (0.6)			
2	146 (20.0)	8 (2.5)			
3	179 (24.5)	98 (30.6)			
4	130 (17.8)	31 (9.7)			
5	87 (11.9)	35 (10.9)			
6	114 (15.6)	142 (44.4)			
Missing	18 (2.5)	4 (1.3)			
**Duration of illness**	12.93 (10.81)	N/A	N/A	N/A	N/A
**Baseline treatment**		N/A	N/A	N/A	N/A
None	23 (3.2)				
Outpatient	355 (48.6)				
Day patient	38 (5.2)				
Inpatient	310 (42.5)				
Missing	4 (0.5)				

^a^The PsyCourse study measures status in the German educational system in detail. In order to make the German educational system comparable to English-speaking systems information on specialized schools, high school and professional education in Germany have been combined to form an ordinal educational scale with “6” being the highest level of education obtained

^b^Age and duration of illness have been reported as mean (standard deviation), while all other categorical variables have been reported as *n* (%). A *t*-test was used for comparison of mean age and *X*^*2*^-tests were used for all categorical comparisons

Socio-demographic information of participants

The correlations between cognitive domains were assessed (Supplementary Fig. 1). Boxplots depicting case versus control performance across all cognitive domains are shown in Supplementary Fig. 2. Investigation of linear models to test the effect of case status on cognitive performance, after adjusting for age and sex, showed a significant effect in the direction expected, i.e., a decreased performance for cases (Supplementary Table 1). Educational attainment was significantly associated with GPS_EDU_ in the direction expected (Supplementary Table 2).

**Fig. 1:**
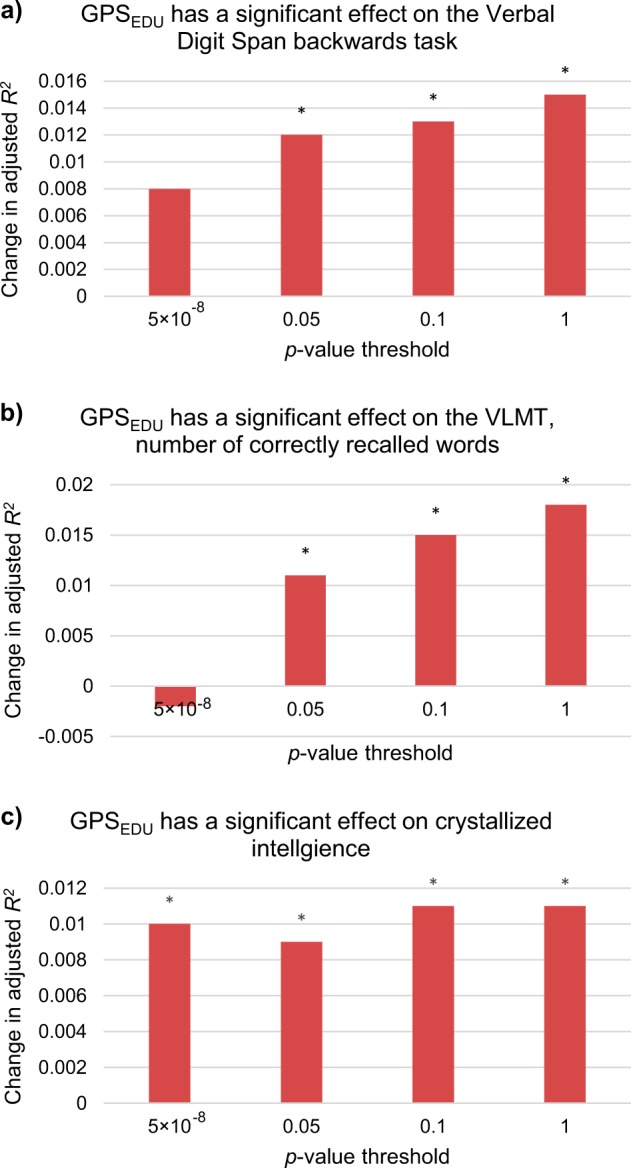
Effect of genome-wide polygenic risk scores for educational attainment (GPS_EDU_) on cognitive performance (Significant *p*-value thresholds were labeled with an Asterix (* *p* < 0.05)). **a** Change in adjusted-*R*^*2*^ after inclusion of GPS_EDU_ in the verbal digit span (backward) model. Baseline model: Adjusted-*R*^*2*^ 0.109; FDR-corrected *p*-values for *p*-value threshold *p*_T_ < 5 × 10^−8^ to 1: 0.050, 0.021, 0.021, 0.021. **b** Change in adjusted-*R*^*2*^ after inclusion of GPS_EDU_ in VLMT (number of correctly recalled words) model. Baseline model: Adjusted-*R*^*2*^ 0.224; FDR-corrected *p*-values for *p*-value threshold *p*_T_ < 5 × 10^−8^ to 1: 0.653, 0.045, 0.025, 0.021. **c** Change in adjusted-*R*^*2*^ after inclusion of GPS_EDU_ in crystallized intelligence (MWT-B) model. Baseline model: Adjusted-*R*^*2*^ 0.214; FDR-corrected *p*-values for *p*-value threshold *p*_T_ < 5 × 10^−8^ to 1: 0.030, 0.031, 0.021, 0.021

**Fig. 2:**
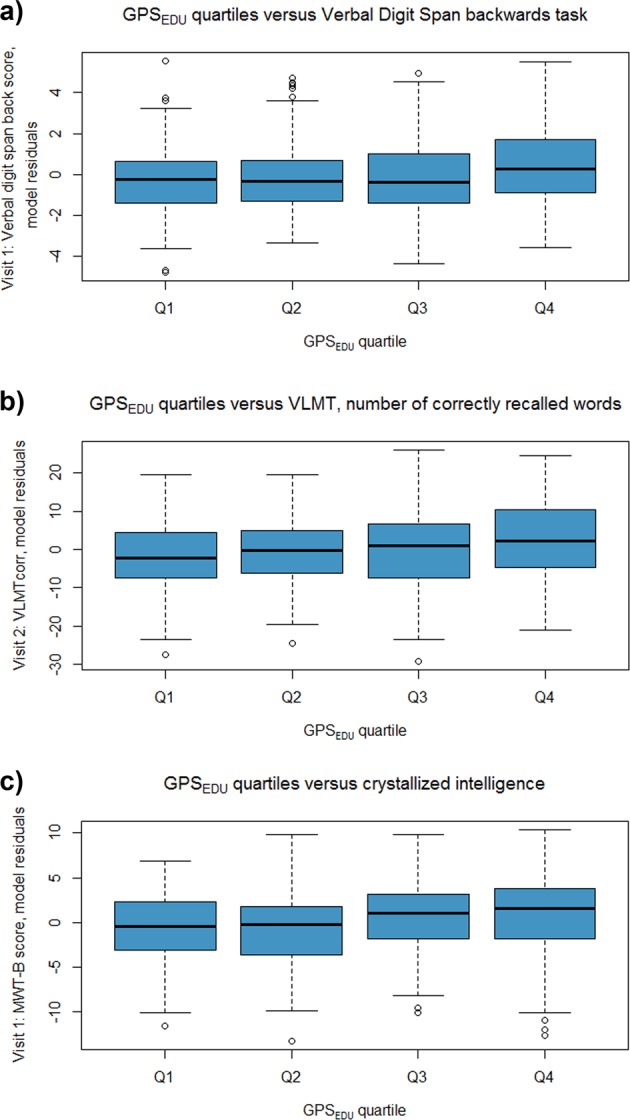
Cognitive performance model residuals plotted against quartiles of genome-wide polygenic risk score for educational attainment (GPS_EDU_). **a** A trend is seen for increased verbal digit span performance with increased load of GPS_EDU_. **b** A trend is seen for increased VLMT performance with increased load of GPS_EDU_. **c** A trend is seen for increased MWT-B performance with increased load of GPS_EDU_

Our investigation of the effect of GPS_EDU_ (*p*_T_ < 1) on cognitive performance in patients resulted in a significant increase in Nagelkerke’s *R*^*2*^ of 1.5% for the verbal digit span (backward; Fig. 1a), 1.9% for the VLMT number of correctly recalled words (Fig. 1b) and 1.1% for crystallized intelligence (Fig. 1c). With more stringent *p*-value thresholds used, i.e., the inclusion of less SNPs, the change in adjusted-*R*^*2*^ decreased. For the verbal digit span (backward) and the VLMT, the GPS_EDU_ based on the *p*-value thresholds *p*_T_ < 0.05, 0.1, and <1 were significant (FDR-adjusted *p* < 0.05; Supplementary Table 3). The score was significant at all *p*-value thresholds for crystallized intelligence (FDR-adjusted *p* < 0.05). The examination of model residuals via quantile–quantile (QQ) plots did not show any extreme deviation from normality (Supplementary Figs. 3–5). Further inspection of model residuals against GPS_EDU_ quartiles showed evidence of increased performance on all three domains with increased GPS_EDU_ scores (Fig. 2). Our results remained robust after correcting for medication (Supplementary Table 4) and diagnosis (Supplementary Table 5). Furthermore, our mediation analysis supports a robust association between GPS_EDU_ and crystallized intelligence that is not mediated by memory parameters (GPS_EDU_ *p* < 0.05; change in adjusted-*R*^*2*^ = 0.0091).

No significant associations between cognitive outcomes and polygenic scores for schizophrenia or bipolar disorder were observed (Supplementary Table 6). Furthermore, neither risk score influenced the significant effects of GPS_EDU_ on the three cognitive domains reported above (Supplementary Table 7). Multicollinearity diagnostics showed no issues of collinearity in our regression analysis (variance inflation factor <5 for all independent variables).

## Discussion

Our study aimed to identify the influence of GPS_EDU_ on several cognitive domains in a transdiagnostic cohort of psychiatric patients. Confirming results of previous research, patients with bipolar disorder and schizophrenia in the PsyCourse cohort performed worse on tests of neurocognitive functioning in comparison to nonclinical controls. In patients, we observed a significant improvement in prediction of cognitive performance with inclusion of GPS_EDU_ for the backward verbal digit span, VLMT (correctly recalled words), and for crystallized intelligence. These findings confirm the ability of GPS_EDU_ to explain variability in linguistic cognitive performance related to working memory and learning in patients with known cognitive deficits. Furthermore, our findings show that cognitive performance measured for these domains were associated with the genetic underpinnings of GPS_EDU_ and not confounded by or associated with GPS_SZ_ or GPS_BD_.

Previous studies have investigated the association between cognitive performance and GPS_EDU_ using summary statistics from an earlier GWAS on educational attainment by Okbay et al.^24^. Our findings compliment earlier evidence supporting an association between cognitive performance and educational attainment, but not schizophrenia genetic risk, in clinical patients. For example, a study by Shafee et al. compared the effect of GPS_SZ_ on three cognitive phenotypes i.e. general cognitive function, premorbid intellectual potential, and years of education completed^30^. The authors found that among healthy individuals, GPS_SZ_ was significantly associated with lower general cognitive functioning, however, found no association between GPS_SZ_ with any cognitive phenotype in patients with psychosis. Furthermore, the authors found significant positive correlations between GPS_EDU_ and both educational attainment and premorbid intelligence in patients with and without psychosis. Another study by Bansal et al. showed GPS_EDU_ could predict 2.09% of variance in premorbid IQ in a large schizophrenia sample^29^. Our findings support earlier suggestions that different cognitive phenotypes vary in their etiologic relationship with schizophrenia and in their genetic overlap with educational attainment^30^. Furthermore, our findings are in line with evidence from the first educational attainment GWAS of 126,559 individuals which identified variants which implicated genes (including *BSN, GBX2, LRRN2, and PIK3C2B*) linked to processes such as learning and long-term memory^27^. These findings are especially interesting given that learning and working memory are among some of the most impaired cognitive process for patients with psychiatric disorders^57^.

While a polygenic score for educational attainment in the general population explained 7–10% of variance in cognitive performance, the score explained at most ~2% in our transdiagnostic cohort^22^. It is difficult to determine whether the smaller effect in our cohort was the result of a different phenotype being measured, i.e., specific cognitive domains and not a composite score, or whether this might reflect the cognitive performance of this unique, transdiagnostic sample being related to other complex genetic-environmental factors. Clearly, future investigations looking at other measures of cognition in large cohorts are warranted. Confounding variables such as acute symptoms may also contribute to the lack of variability explained in this case, although we have tried to capture this by controlling for current in/outpatient status and symptom severity. Furthermore, although based on big samples, polygenic scores “may not be sufficiently powerful to capture signs of disrupted neurodevelopment” in these patients as they exclude rare copy number variations and deleterious exonic mutations which may have important consequences^52^.

On both a phenotypic and genetic level, intelligence has been associated with psychiatric disorders. For example, individuals with a level of intelligence one standard deviation below the mean, have ~60% higher risk of hospitalization for schizophrenia^58^. There is also evidence supporting an association between poorer school performance and higher risk for schizophrenia^52^. In addition, several longitudinal studies have linked deficits in premorbid IQ with subsequent schizophrenia development, which was also shown for mood disorders^52,59^. The evidence, however, linking intelligence and affective disorders has been more inconsistent. For example, bipolar disorder has been associated with higher childhood IQ and an increased genetic risk of bipolar disorder has been associated with creativity and higher education^60–62^. However, no such associations have been reported by studies of adolescent or adult IQ^60^. Nevertheless, there are known genetic variants influencing both intelligence and psychiatric disorders which, in part, explain the phenotypic link between intelligence and these disorders^58^.

We investigated the potential influence of GPS_SZ_ and GPS_BD_ on cognitive performance. These relationships seem to be complex and while the genetic overlap between schizophrenia susceptibility with cognitive performance has been widely investigated in the literature with conflicting findings, less has been done in bipolar disorder^8,63–65^. The lack of an association observed between either the GPS_SZ_ or GPS_BD_ with cognitive performance in our study emphasizes several issues inherent to these types of investigations. The first is that GPS_EDU_ is based on a much larger discovery sample than GPS_SZ_ and GPS_BD_, meaning GPS_EDU_ had higher statistical power to capture smaller effect sizes and more accurate estimates for single SNPs of which the score is based on. Presuming the most optimistic estimate for variance explained in cognitive performance by the GPS_SZ_ of 1.6% that has previously been reported^8^, a sample of ~500 participants would be required to drive the effect of schizophrenia genetic risk scores on cognitive performance. However, given a more conservative estimate of 0.3%^66^ variance explained, a sample size of over 2 600 participants would be required, suggesting that power may indeed be an issue in our study (Supplementary Fig. 6). This is also true with regards to GPS_BD_ in which genetic effects are likely to be at least as subtle. This again highlights the value of analyzing a proxy-phenotype such as educational attainment.

The second issue is in relation to the cognitive domains that were analyzed. As studies often use different cognitive tests from the wide variety that are available, it could be that the genetic risk of schizophrenia and bipolar disorder are more closely linked to domains that went unmeasured in our study. Perhaps if we had used a composite score across all domains or different neurocognitive tests in general, a significant effect would have been observed. Unfortunately, due to the longitudinal nature of our study which led to missing data across the different cognitive outcomes tested, a composite score analysis with adequate power was not feasible.

Our findings should be considered in light of a few limitations. The first is that our patients represent a chronic sample of heterogeneously treated patients. As these patients have been prescribed a wide range of medications at different dosages, correcting for the possible influence of medication is not an easy task. Not knowing how different drugs might interact with or influence cognition throughout the course of the disorder is a limitation that always must be considered in psychiatric research, and this problem has yet to have a perfect solution. A second limitation of our study is generalizability considering we investigated raw scores for several cognitive domains. As mentioned above, one of the major issues in the field at this time is the complexity in measuring this phenotype and with a plethora of tests that can be used, it is difficult to say how generalizable our findings are to other cognitive tests within the same cognitive domain in different cohorts. For example, while crystallized intelligence was measured, our study failed to consider fluid intelligence which has a higher heritability component than crystallized intelligence^67^. It is also important to note that while executive functions are related, they are also diverse^68^. While the TMT used in our study is a measure of task switching, other executive functions like the updating process of working memory and inhibition should be explored. Lastly, we must acknowledge that our study has only assessed linguistic memory and not visuospatial memory. As these are two unique types of memory^69^, future investigations are warranted to determine how the two might differ in association with GPS_EDU_.

Although remarkable heterogeneity of cognitive deficits exists among individuals with psychiatric disorders, in general these deficits are, by a moderate degree, less severe in chronic bipolar patients in comparison to chronic schizophrenia patients. Furthermore, the trajectories of these impairments are quite different^70^. Often, cognitive deficits are apparent before the onset of disease in individuals with schizophrenia^71^. Approximately 70% of bipolar patients exhibit cognitive deficits, especially related to verbal memory and attention^57^, which often manifest in young adults^60^. Despite these known differences for bipolar disorder and schizophrenia, we did not observe a significant effect of diagnosis on the effect of GPS_EDU_ related to cognitive performance. These diagnostic differences were most likely captured by the PANSS sum scores included in our models, which was highly significant. Evidence also supports an increase in the heritable component of intelligence with age^72^. Considering this knowledge, future studies, longitudinal in design, would be highly beneficial. It would be intriguing to see how the polygenic score for educational attainment can explain variability in cognitive performance throughout the course of the disorder. While our sample consisted of chronic mid-aged patients in which cognitive performance was rather stable across visits, it would be valuable to investigate younger cohorts of patients, even before the onset of disease, to determine how instability in cognitive performance throughout the disease course might influence the association between GPS_EDU_ and cognition. This would help determine at which points the underlying genetic components are most influential and help identify at which periods environmental influences might be more prominent in determining cognitive abilities.

## Conclusions

Identifying a genetic component related to distinct neurocognitive profiles has potential to identify a more burdened subgroup of patients that in turn might be at risk for lower levels of functioning and poor social outcomes. This sort of information targets patients for more personalized interventions^73,74^. Here we have explained only a small fraction of variance in cognitive performance in patients with psychiatric disorders using the genetic variants associated with educational attainment. These findings highlight the importance of other uncaptured environmental exposures that have major influences on cognitive abilities and ultimately levels of functioning in these patients. Future studies, over the course of the disorder, would be informative to determine how this association changes over time, and at which periods environment may play the most influential role^60^. Furthermore, future studies should factor in the complex pleiotropic relationships between these traits to generate enhanced polygenic scores to further clarify their genetic architecture^75^. Moreover, hypothesis-based polygenic scores could help uncover biological pathways related to cognitive performance.

## Supplementary information

Supplementary Material
