# Supplementary material for: The genetic relationship between educational attainment and cognitive performance in major psychiatric disorders
Source: Transl Psychiatry. 2019 Aug 28;9:210. doi: 10.1038/s41398-019-0547-x (PMC6713703; doi:10.1038/s41398-019-0547-x)
Supplement: Supplementary file 1 — Supplementary Material [file 41398_2019_547_MOESM1_ESM.docx]

**Supplementary Material:** The genetic relationship between educational attainment and cognitive performance in major psychiatric disorders


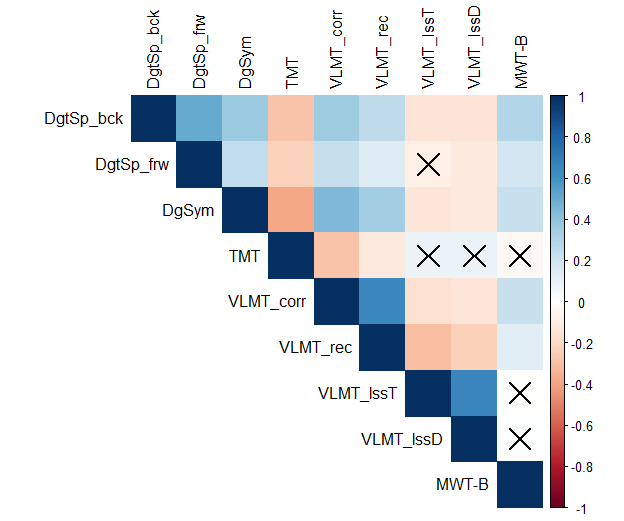


**Figure S1.** Pairwise correlations between cognitive outcomes in cases only. Shown are Pearson correlation coefficients, insignificant correlations labeled with “X” (uncorrected p > 0.05). Note: DgtSp_bck – Verbal digit span (backwards task); DgtSp_frw – Verbal digit span (forwards task); DgSym – Digit symbol test; TMT – TMT reaction time difference; VLMT_corr – VLMT number of correctly recalled words; VLMT_lssT – VLMT loss of recalled words after time; VLMT_lssD – VLMT loss of recalled words after distraction; VLMT_rec – VLMT number of correctly recognized words


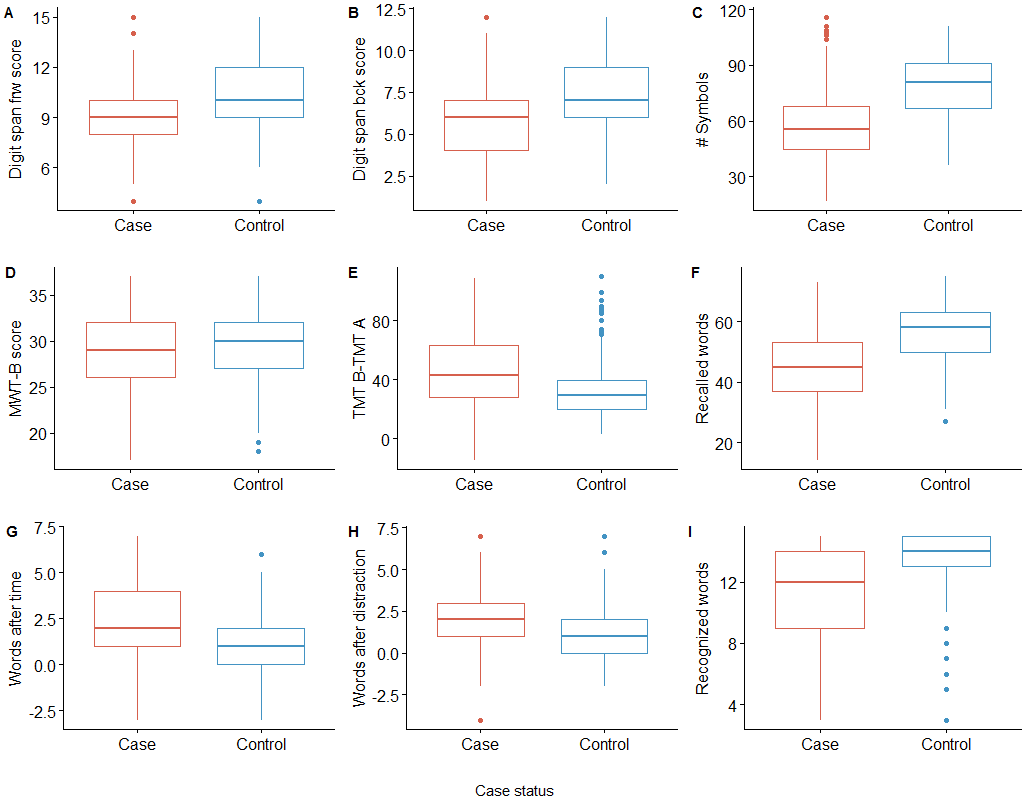


**Figure S2.** Boxplots depicting the distribution of cognitive performance for cases and controls across all cognitive domains**. A.** Verbal digit span, forward task score **B.**  Verbal digit span, backward task score **C.** Digit symbol test, number of correct symbols **D.** MWT-B score **E.** Trail-Making-Test (TMT), difference in reaction time (TMT B-TMT A) **F.**  Verbal Learning and Memory Test (VLMT): number of correctly recalled words **G.** VLMT: number of words lost after time **H.** VLMT: number of words lost after distraction **I.**  VLMT: number of correctly recognized word

**Table S1.** The effect of case status on cognitive performance

| Outcome | Cases  mean (sd) | Controls  mean (sd) | Estimate | Std.  Error | T-value | *p*-value |  |
| --- | --- | --- | --- | --- | --- | --- | --- |
| Visit 1 |  |  |  |  |  |  |  |
| TMT reaction time difference | 47.78 (24.80) | 32.10 (17.91) | -12.55 | 1.49 | -8.43 | < 2.00×10^-16^ *** |  |
| Digit symbol test | 56.37 (15.79) | 79.03 (15.91) | 18.39 | 1.03 | 17.86 | < 2.00×10^-16^ *** |  |
| Verbal digit span backwards task | 5.72 (1.95) | 7.25 (2.14) | 1.40 | 0.14 | 9.87 | < 2.00×10^-16^ *** |  |
| Verbal digit span forwards task | 9.01 (1.94) | 10.32 (2.06) | 1.18 | 0.15 | 8.15 | 1.13×10^-15^ *** |  |
| MWT-B (crystallized intelligence) | 28.34 (4.56) | 29.63 (3.61) | 1.67 | 0.33 | 5.05 | 5.90×10^-7 ***^ |  |
| Visit 2 |  |  |  |  |  |  |  |
| VLMT: correctly recalled words | 44.99 (11.57) | 56.35 (9.24) | 8.87 | 0.83 | 10.70 | < 2×10^-16^ *** |  |
| VLMT: loss of words after time | 2.21 (2.01) | 1.22 (1.77) | -0.80 | 0.16 | -5.02 | 6.81×10^-07^ *** |  |
| VLMT: loss of words after distraction | 2.07 (1.82) | 1.26 (1.65) | -0.66 | 0.15 | -4.41 | 1.21×10^-05^ *** |  |
| VLMT: correctly recognized words | 11.28 (3.46) | 13.44 (2.13) | 1.45 | 0.23 | 6.20 | 1.06×10^-09^ *** |  |
| *Note:* Effect estimates and associated standard errors, T-values and *p*-values adjusted for age and sex | | | | | | | |

**Table S2.** The effect of GPS_EDU_ quartile on level of educational attainment in cases, adjusting for age, sex, the interaction between age and sex, and the first 10 principle components

|  | **Proportional odds ratio** | **95% CI** | ***p*-value** |
| --- | --- | --- | --- |
| GPS_EDU_ quartile 2 | 1.478 | 1.013 - 2.159 | 0.043* |
| GPS_EDU_ quartile 3 | 1.794 | 1.237 - 2.606 | 0.002* |
| GPS_EDU_ quartile 4 | 2.495 | 1.706 - 3.657 | 2.56×10^-6^*** |
| Age (years) | 0.866 | 0.695 - 1.078 | 0.200 |
| Sex (male) | 0.728 | 0.555 - 0.956 | 0.022* |
| PC1 | 1.138 | 1.002 - 1.303 | 0.046* |
| PC2 | 1.137 | 0.995 - 1.300 | 0.057 |
| PC3 | 0.910 | 0.787 - 1.048 | 0.192 |
| PC4 | 1.005 | 0.857 - 1.174 | 0.952 |
| PC4 | 0.957 | 0.810 - 1.110 | 0.571 |
| PC6 | 1.101 | 0.960 - 1.263 | 0.169 |
| PC7 | 0.970 | 0.850 - 1.107 | 0.653 |
| PC8 | 0.896 | 0.785 - 1.022 | 0.102 |
| PC9 | 0.981 | 0.859 - 1.120 | 0.778 |
| PC10 | 1.003 | 0.882 - 1.141 | 0.959 |
| Age (years):Sex (M) | 1.952 | 1.473 - 2.590 | 3.36×10^-6^*** |
| * *p* < 0.05 ** *p* < 0.005 *** *p* < 0.005 | | | |

**Table S3.** Effect of GPS_EDU_ on cognitive performance models adjusted for age, age^2^, sex, in/outpatient status, center, PANSS sum scores, principle components

| **Model** | **GPS**  ***p-*value threshold** | ***p*-value** | **FDR corrected**  ***p*-value** | **Base model adjusted *R^2^*** | **Adjusted *R^2^* after GPS_EDU_ inclusion** |
| --- | --- | --- | --- | --- | --- |
| **Visit 1** |  |  |  |  |  |
| V1: Verbal digit span backwards task |  |  |  | 0.109 |  |
|  | 1 | 0.001 | 0.021 |  | 0.124 |
|  | 0.1 | 0.002 | 0.021 |  | 0.122 |
|  | 0.05 | 0.004 | 0.021 |  | 0.121 |
|  | 5×10^-8^ | 0.015 | 0.050 |  | 0.117 |
| V1: Verbal digit span forwards task |  |  |  | 0.066 |  |
|  | 1 | 0.477 | 0.553 |  | 0.066 |
|  | 0.1 | 0.313 | 0.470 |  | 0.066 |
|  | 0.05 | 0.384 | 0.522 |  | 0.065 |
|  | 5×10^-8^ | 0.435 | 0.553 |  | 0.065 |
| V1: Digit symbol test |  |  |  | 0.292 |  |
|  | 1 | 0.206 | 0.417 |  | 0.293 |
|  | 0.1 | 0.241 | 0.429 |  | 0.293 |
|  | 0.05 | 0.469 | 0.553 |  | 0.292 |
|  | 5×10^-8^ | 0.765 | 0.787 |  | 0.291 |
| V1: MWT-B |  |  |  | 0.214 |  |
|  | 1 | 0.003 | 0.021 |  | 0.225 |
|  | 0.1 | 0.003 | 0.021 |  | 0.225 |
|  | 0.05 | 0.008 | 0.030 |  | 0.223 |
|  | 5×10^-8^ | 0.007 | 0.030 |  | 0.224 |
| V1: TMT B-TMT A |  |  |  | 0.091 |  |
|  | 1 | 0.454 | 0.464 |  | 0.091 |
|  | 0.1 | 0.611 | 0.417 |  | 0.090 |
|  | 0.05 | 0.560 | 0.417 |  | 0.090 |
|  | 5×10^-8^ | 0.391 | 0.417 |  | 0.091 |
| V2: VLMT correctly recalled words |  |  |  | 0.224 |  |
|  | 1 | 0.002 | 0.021 |  | 0.243 |
|  | 0.1 | 0.005 | 0.025 |  | 0.239 |
|  | 0.05 | 0.013 | 0.045 |  | 0.235 |
|  | 5×10^-8^ | 0.617 | 0.652 |  | 0.222 |
| V2: VLMT loss of words after time |  |  |  | 0.050 |  |
|  | 1 | 0.283 | 0.463 |  | 0.050 |
|  | 0.1 | 0.3075 | 0.522 |  | 0.049 |
|  | 0.05 | 0.296 | 0.464 |  | 0.050 |
|  | 5×10^-8^ | 0.193 | 0.417 |  | 0.052 |
| V2: VLMT loss of words after distraction |  |  |  | 0.015 |  |
|  | 1 | 0.199 | 0.417 |  | 0.017 |
|  | 0.1 | 0.220 | 0.417 |  | 0.017 |
|  | 0.05 | 0.187 | 0.417 |  | 0.017 |
|  | 5×10^-8^ | 0.035 | 0.104 |  | 0.025 |
| V2: VLMT correctly recognized words |  |  |  | 0.142 |  |
|  | 1 | 0.199 | 0.417 |  | 0.144 |
|  | 0.1 | 0.210 | 0.417 |  | 0.144 |
|  | 0.05 | 0.250 | 0.429 |  | 0.143 |
|  | 5×10^-8^ | 0.936 | 0.936 |  | 0.139 |


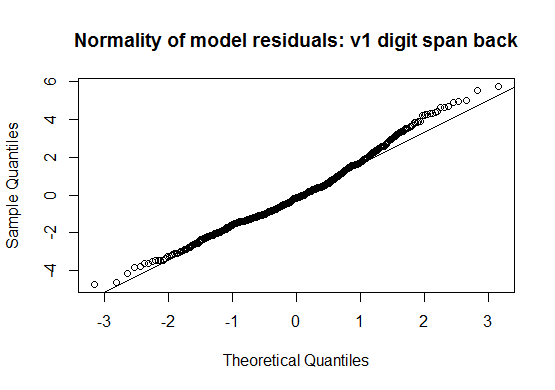


**Figure S3**. QQ plot for visual inspection of normality of model residuals – Visit 1 verbal digit span backward model


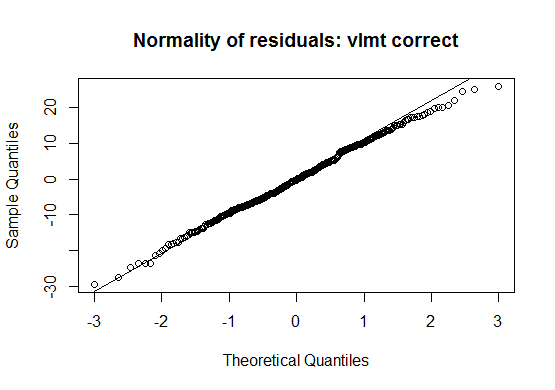


**Figure S4.** QQ plot for visual inspection of normality of residuals- Visit 2 VLMT correctly recalled words model


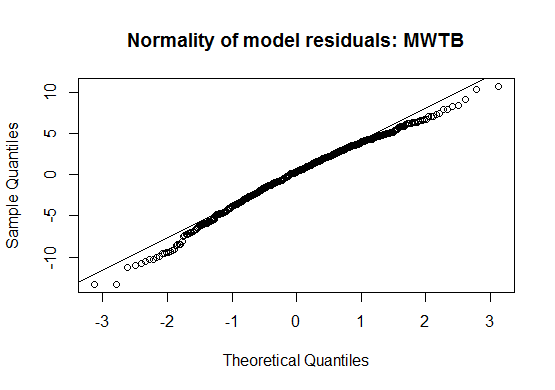


**Figure S5**. QQ plot for visual inspection of normality of residuals- Visit 1 MWT-B (crystallized intelligence) model

**Table S4**. Effect of GPS_EDU_ on cognitive performance models adjusted for age, age^2^, sex, in/outpatient status, center, PANSS sum scores, principle components, and **medication**

| **Model** | **GPS**  ***p*-value threshold** | ***p*-value** | **FDR corrected *p*-value** | **Base model adjusted *R^2^*** | **Adjusted *R^2^* after GPS_EDU_ inclusion** |
| --- | --- | --- | --- | --- | --- |
| V1: Verbal digit span backwards task |  |  |  | 0.109 |  |
|  | 1 | 0.001 | 0.010 |  | 0.124 |
|  | 0.1 | 0.002 | 0.010 |  | 0.123 |
|  | 0.05 | 0.003 | 0.014 |  | 0.121 |
|  | 5×10^-8^ | 0.012 | 0.041 |  | 0.117 |
| V1: Verbal digit span forwards task |  |  |  | 0.066 |  |
|  | 1 | 0.387 | 0.478 |  | 0.066 |
|  | 0.1 | 0.242 | 0.396 |  | 0.067 |
|  | 0.05 | 0.300 | 0.446 |  | 0.066 |
|  | 5×10^-8^ | 0.390 | 0.478 |  | 0.066 |
| V1: Digit symbol test |  |  |  | 0.301 |  |
|  | 1 | 0.184 | 0.346 |  | 0.302 |
|  | 0.1 | 0.202 | 0.346 |  | 0.302 |
|  | 0.05 | 0.398 | 0.478 |  | 0.301 |
|  | 5×10^-8^ | 0.747 | 0.767 |  | 0.300 |
| V1: MWT-B |  |  |  | 0.213 |  |
|  | 1 | 0.001 | 0.010 |  | 0.226 |
|  | 0.1 | 0.002 | 0.010 |  | 0.226 |
|  | 0.05 | 0.004 | 0.015 |  | 0.224 |
|  | 5×10^-8^ | 0.006 | 0.020 |  | 0.223 |
| V1: TMT B-TMT A |  |  |  | 0.098 |  |
|  | 1 | 0.526 | 0.592 |  | 0.097 |
|  | 0.1 | 0.680 | 0.720 |  | 0.097 |
|  | 0.05 | 0.623 | 0.680 |  | 0.097 |
|  | 5×10^-8^ | 0.388 | 0.478 |  | 0.098 |
| V2: VLMT correctly recalled words |  |  |  | 0.253 |  |
|  | 1 | 0.001 | 0.010 |  | 0.276 |
|  | 0.1 | 0.001 | 0.010 |  | 0.273 |
|  | 0.05 | 0.004 | 0.015 |  | 0.269 |
|  | 5×10^-8^ | 0.465 | 0.540 |  | 0.252 |
| V2: VLMT loss of words after time |  |  |  | 0.046 |  |
|  | 1 | 0.290 | 0.446 |  | 0.047 |
|  | 0.1 | 0.391 | 0.478 |  | 0.046 |
|  | 0.05 | 0.310 | 0.446 |  | 0.046 |
|  | 5×10^-8^ | 0.141 | 0.338 |  | 0.050 |
| V2: VLMT loss of words after distraction |  |  |  | 0.014 |  |
|  | 1 | 0.182 | 0.346 |  | 0.016 |
|  | 0.1 | 0.196 | 0.346 |  | 0.016 |
|  | 0.05 | 0.166 | 0.346 |  | 0.017 |
|  | 5×10^-8^ | 0.022 | 0.066 |  | 0.027 |
| V2: VLMT correctly recognized words |  |  |  | 0.148 |  |
|  | 1 | 0.125 | 0.321 |  | 0.152 |
|  | 0.1 | 0.125 | 0.321 |  | 0.152 |
|  | 0.05 | 0.151 | 0.340 |  | 0.151 |
|  | 5×10^-8^ | 0.859 | 0.859 |  | 0.146 |

**Table S5.** Effect of GPS_EDU_ on cognitive performance models adjusted for age, age^2^, sex, in/outpatient status, center, PANSS sum scores, principle components, and **diagnosis**

| **Model** | **GPS**  ***p*-value threshold** | ***p*-value** | **FDR corrected *p*-value** | **Base model adjusted *R^2^*** | **Adjusted *R^2^* after GPS_EDU_ inclusion** |
| --- | --- | --- | --- | --- | --- |
| V1: Verbal digit span backwards task |  |  |  | 0.106 |  |
|  | 1 | 0.001 | 0.018 |  | 0.121 |
|  | 0.1 | 0.002 | 0.018 |  | 0.119 |
|  | 0.05 | 0.003 | 0.018 |  | 0.118 |
|  | 5×10^-8^ | 0.015 | 0.050 |  | 0.114 |
| V1: Verbal digit span forwards task |  |  |  | 0.071 |  |
|  | 1 | 0.419 | 0.525 |  | 0.070 |
|  | 0.1 | 0.262 | 0.450 |  | 0.071 |
|  | 0.05 | 0.342 | 0.492 |  | 0.071 |
|  | 5×10^-8^ | 0.365 | 0.506 |  | 0.071 |
| V1: Digit symbol test |  |  |  | 0.302 |  |
|  | 1 | 0.233 | 0.441 |  | 0.302 |
|  | 0.1 | 0.290 | 0.474 |  | 0.302 |
|  | 0.05 | 0.548 | 0.616 |  | 0.302 |
|  | 5×10^-8^ | 0.725 | 0.746 |  | 0.302 |
| V1: MWT-B |  |  |  | 0.214 |  |
|  | 1 | 0.003 | 0.018 |  | 0.226 |
|  | 0.1 | 0.003 | 0.018 |  | 0.226 |
|  | 0.05 | 0.006 | 0.026 |  | 0.224 |
|  | 5×10^-8^ | 0.006 | 0.026 |  | 0.224 |
| V1:TMT B-TMT A |  |  |  | 0.102 |  |
|  | 1 | 0.448 | 0.537 |  | 0.101 |
|  | 0.1 | 0.603 | 0.638 |  | 0.100 |
|  | 0.05 | 0.564 | 0.616 |  | 0.100 |
|  | 5×10^-8^ | 0.409 | 0.525 |  | 0.101 |
| V2: VLMT correctly recalled words |  |  |  | 0.226 |  |
|  | 1 | 0.002 | 0.018 |  | 0.246 |
|  | 0.1 | 0.004 | 0.019 |  | 0.242 |
|  | 0.05 | 0.011 | 0.039 |  | 0.238 |
|  | 5×10^-8^ | 0.559 | 0.616 |  | 0.224 |
| V2: VLMT loss of words after time |  |  |  | 0.054 |  |
|  | 1 | 0.316 | 0.492 |  | 0.054 |
|  | 0.1 | 0.423 | 0.525 |  | 0.053 |
|  | 0.05 | 0.331 | 0.492 |  | 0.054 |
|  | 5×10^-8^ | 0.192 | 0.441 |  | 0.056 |
| V2: VLMT loss of words after distraction |  |  |  | 0.015 |  |
|  | 1 | 0.204 | 0.441 |  | 0.017 |
|  | 0.1 | 0.231 | 0.441 |  | 0.017 |
|  | 0.05 | 0.216 | 0.441 |  | 0.017 |
|  | 5×10^-8^ | 0.037 | 0.111 |  | 0.025 |
| V2: VLMT correctly recognized words |  |  |  | 0.138 |  |
|  | 1 | 0.207 | 0.441 |  | 0.140 |
|  | 0.1 | 0.222 | 0.441 |  | 0.140 |
|  | 0.05 | 0.261 | 0.450 |  | 0.139 |
|  | 5×10^-8^ | 0.893 | 0.893 |  | 0.135 |

**Table S6.** Effect of GPS_BD_ and GPS_SZ_ on cognitive performance, adjusted for age, age^2^, sex, in/outpatient status, center, PANSS sum scores, and principle components

| Model | Base model adjusted- *R^2^* | *p*-value GPS_SZ_ | Adjusted *R^2^* after GPS_SZ_ inclusion | *p-*value GPS_BD_ | Adjusted-*R^2^* after GPS_BD_ inclusion |
| --- | --- | --- | --- | --- | --- |
| V1: Verbal digit span backwards task | 0.109 | 0.268 | 0.109 | 0.312 | 0.109 |
| V1: Verbal digit span forwards task | 0.067 | 0.373 | 0.067 | 0.399 | 0.067 |
| V1: Digit symbol test | 0.291 | 0.838 | 0.290 | 0.786 | 0.290 |
| V1: MWT-B | 0.214 | 0.436 | 0.214 | 0.498 | 0.214 |
| V1: TMT B-TMT A | 0.093 | 0.174 | 0.094 | 0.208 | 0.094 |
| V2: VLMT correctly recalled words | 0.223 | 0.363 | 0.222 | 0.531 | 0.221 |
| V2: VLMT words lost after time | 0.038 | 0.399 | 0.037 | 0.050 | 0.046 |
| V2: VLMT words lost after distraction | 0.019 | 0.826 | 0.016 | 0.456 | 0.018 |
| V2: VLMT correctly recognized words | 0.143 | 0.60 | 0.150 | 0.965 | 0.140 |

**Table S7.** Effect of GPS_BD_ and GPS_SZ_ on the association between GPS_EDU_ and cognitive performance

| Model | *p*-value GPS_EDU_ after adjusting for GPS_SZ_^a^ | Change in adjusted-*R^2^* after GPS_EDU_ inclusion^a^ | *p*-value GPS_EDU_ after adjusting for GPS_BD_^b^ | Change in adjusted-*R^2^* after GPS_EDU_ inclusion^b^ |
| --- | --- | --- | --- | --- |
| V1: Verbal digit span backwards task | 0.002 | 0.013 | 0.001 | 0.014 |
| V1: MWT-B | 0.004 | 0.011 | 0.003 | 0.011 |
| V2: VLMT correctly recalled words | 0.002 | 0.020 | 0.002 | 0.019 |
| ^a^ Adjusting for age, age^2^, sex, in/outpatient status, center, PANSS sum scores, principle components and GPS_SZ_  ^b^ Base model including age, age^2^, sex, in/outpatient status, center, PANSS sum scores, principle components and GPS_BD_ | | | | |


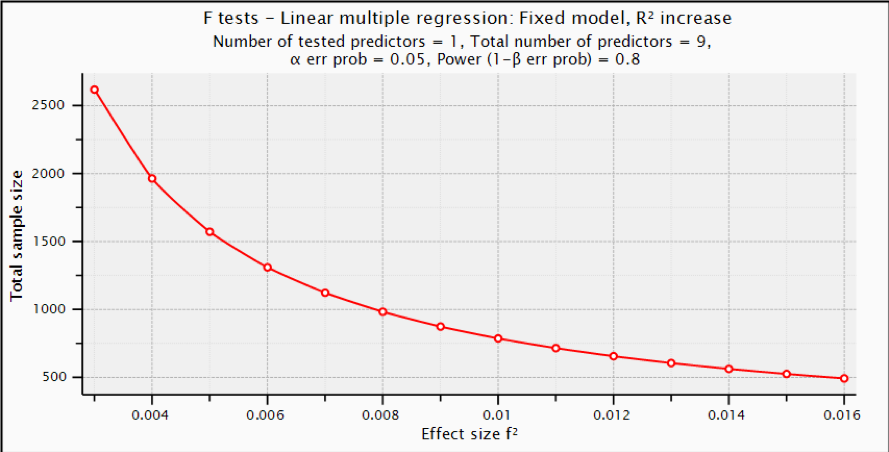


**Figure S6.** Post-hoc power calculation using the freely available G* Power 3.1^1, 2^. Plot depicting required sample sizes required to drive the effect of GPS_SZ_ on cognitive performance to significance according to a range of effect sizes (change in adjusted R^2^ between a model including only covariates and a model including covariates and GPS_SZ_). Note: Alpha level 0.05, 80% power, number of tested predictors = 1, total number of predictors = 9.

**References**

1. Faul, F., Erdfelder, E., Lang, A.-G. & Buchner, A. G*Power 3: A flexible statistical power analysis program for the social, behavioral, and biomedical sciences. *Behav. Res. Methods* 2007; **39**(2)**:** 175-191.

2. Faul, F., Erdfelder, E., Buchner, A. & Lang, A.-G. Statistical power analyses using G* Power 3.1: Tests for correlation and regression analyses. *Behav. Res. Methods* 2009; **41**(4)**:** 1149-1160.
